# Top 10 reasons your manuscript may be rejected without review

**DOI:** 10.1186/s13071-022-05543-w

**Published:** 2022-11-10

**Authors:** Filipe Dantas-Torres

**Affiliations:** Aggeu Magalhães Institute, Oswaldo Cruz Foundation (Fiocruz), Recife, PE Brazil

Scientific writing and publishing have evolved considerably in the past century. This is partly due to changes in the way we think, design and do research. Technological advancements have also played a major role in this process. Not long ago, submissions used to be made by mail. I remember going to a courier service office to submit a hard copy of my manuscript along with a compact disk containing the figures. Electronic systems have simplified the submission process considerably, reducing turnaround times and making it easy to follow up the whole peer review process. Believe me, it used to be more difficult than it is now.

Nowadays, the number of submissions received by a leading journal in its field may be well above 1000 per year, which poses several challenges to editors and journal staff. Probably the biggest challenge for these journals these days is finding available reviewers. With the unstoppable growth of the publishing industry, the number of new publishers and journals launched each year is scary. As a result, senior researchers are receiving numerous review invitations weekly or even daily.

Very often, editors need to send out invitations to more than 10 potential reviewers to secure the one or two who will actually agree to review the manuscript. Of these, some will never submit a report. This is a huge problem, and there is no easy long-term solution. Some publishers are trying to find solutions by, for example, offering incentives to reviewers, including certificates and discounts in article processing charges for those who eventually decide to submit a manuscript to the very same journal. While this may have a positive impact in terms of the percentage of reviewers who agree to review a manuscript, the overall impact of this on the peer review process is little known.

Another strategy some journals adopt to reduce the burden on journal staff, editors and reviewers is being more selective in deciding which manuscripts should actually be sent out for peer review. As an example, the *JAMA Internal Medicine* reported that 78% of the manuscripts received in 2017 were rejected without review [1]. The current pre-review rejection rate of *Parasites & Vectors* (*P&V*) is 39%. Common reasons for rejection without peer review in *P&V* are listed in Box 1.

In 2022, for the first time, *P&V*’s impact factor surpassed the mark of 4. There is an increasing trend in the number of manuscripts received by *P&V*, not only due to the new impact factor, but because *P&V* continues to stand out as one of the leading journals in fields of parasitology and tropical medicine. As a response to the increasing number of submissions, we are inviting new editors and discussing strategies to improve the benefits of both editors and reviewers, to whom I would like to express my gratitude for their invaluable contributions to *P&V*.

Our team of editors will keep working tirelessly to ensure a rapid peer review process as far as possible. Nonetheless, in a time when short messages and videos are the rule, it is important to remember that speed does not mean quality, especially in the context of important issues, such as scientific publishing.

Box 1. Common reasons why submissions are rejected without peer review **Out of scope of the journal**: Many submissions are not sent out for peer review simply because they are out of scope of the journal. If you are unsure whether your manuscript fits the scope of the journal, send a pre-submission inquiry beforehand. If your manuscript was already rejected for this reason, look for a more suitable journal.

**Local interest**: Many studies are rejected because the results and conclusions are of local interest. A recurrent example are small-scale cross-sectional studies of local interest. While choosing a journal, ask yourself whether your research is of local or broad interest. Sometimes, when your manuscript is rejected for its local interest, you can take another look at your data to see whether it can be used to investigate a new hypothesis of broad interest. This will largely depend on your study design and other methodological factors that may or may not allow you to investigate a new hypothesis using a different analysis. If this is unfeasible, which is usually the case, simply look for a more regional journal.

**No novelty**: Some studies are well designed and conducted, but are limited in terms of novelty. While planning a study, make sure you have a strong research question which is relevant to the field. Once you have a novel hypothesis, double check if it is really new by performing a comprehensive literature review in different databases. It is important not only to search in PubMed and other large databases (e.g. Embase, Cochrane Library, Scopus, Web of Science), but also in more regional databases. In this regard, WHO maintains the Global Index Medicus (GIM), which provides access to biomedical and public health literature from regional databases, including the African Index Medicus (AIM), the Scientific and Technical Literature of Latin America and the Caribbean (LILACS), Index Medicus for Eastern Mediterranean Region (IMEMR), Index Medicus for South-East Asia Region (IMSEAR) and the Western Pacific Region Index Medicus (WPRIM).

**Inadequate study design and procedures**: The study design may be inappropriate for answering your hypothesis, including small sample size, lack of randomization and proper controls. You may also have used an old methodology that has been surpassed by more powerful methods which could provide more robust results.

**Incomplete methodological details and data reporting**: All readers (editors, reviewers and journal readership) must be able to fully understand and repeat your experiments and analyses. Complete data reporting is also pivotal. Make sure you are reporting all methodological details and all relevant data, including complete statistical results (*P*-value alone is not enough).

**Data availability issues**: All data that support the findings and conclusions of the study should be included in the published manuscript, its additional files and/or in an electronic repository. Moreover, new DNA sequences generated in your study should be deposited in GenBank, and accession numbers must be provided. In the data availability statement, sentences like “Data are available from the corresponding author upon reasonable request” should be avoided. Unless the corresponding author has superpowers and is immortal, she/he will not live forever, which means that in the future data will no longer be available.

**Ethical issues**: If your research involve humans or animals, you must obtain an informed consent and an approval from an ethics committee. Also, make sure you follow the three Rs (3Rs) principle. Other necessary authorizations (e.g. for animal trapping or for the use of genetically modified organisms) must also be obtained and provided in the manuscript.

**Poor presentation**: Manuscripts will not be rejected due to minor formatting issues that can be corrected during production. However, manuscripts lacking proper structure, those which are badly formatted and/or those with low-quality figures may be rejected. Make sure your article conforms with the journal style guide and that your figures are of high quality (not pixelated or blurry). If you do not know how to prepare high-quality figures, seek technical support before submitting your manuscript. Some tips on how to prepare good figures are available in the online* P&V* editorial style guide [2].

**Poor English**: English writing is a major obstacle for non-English authors, particularly from institutions that do not offer translation and/or English editing services. Nonetheless, manuscripts written in poor English, which cannot be understood by readers (editors, reviewers and journal readership) will not be sent out for review.

**Plagiarism**: All manuscripts submitted to our journal are automatically checked for plagiarism. Our similarity checking tool (CrossRef, powered by iThenticate) will highlight manuscripts with high similarity values. While I am writing this section, I just received a new submission with a 52% overall similarity (31% similarity with a single paper published from the very same authors). Self-plagiarism is also plagiarism, so make sure you modify your sentences and cite all necessary references while writing your manuscript.

## References

[1] Redberg RF (2018). JAMA internal medicine-the year in review, 2017. JAMA Intern Med.

[2] Springer Nature. Parasites & Vectors. 2020. Submission guidelines. https://parasitesandvectors.biomedcentral.com/submission-guidelines. Accessed 8 Oct 2022.

